# Real-time sensing of neurotransmitters by functionalized nanopores embedded in a single live cell

**DOI:** 10.1186/s43556-021-00026-3

**Published:** 2021-02-28

**Authors:** Xialin Zhang, Linqin Dou, Ming Zhang, Yu Wang, Xin Jiang, Xinqiong Li, Long Wei, Yuejia Chen, Cuisong Zhou, Jia Geng

**Affiliations:** 1 Department of Laboratory Medicine, State Key Laboratory of Biotherapy and Cancer Center, West China Hospital, Sichuan University and Collaborative Innovation Center, Chengdu, 610041 China; 2 College of Chemistry, Sichuan University, Chengdu, 610041 China

**Keywords:** Nanopore, Neurotransmitter detection, Artificial synapse, Biosensor

## Abstract

Interface between neuron cells and biomaterials is the key to real-time sensing, transmitting and manipulating of neuron activities, which are the long-term pursue of scientists and gain intense research focus recently. It is of great interest to develop a sensor with exquisite sensitivity and excellent selectivity for real-time monitoring neurotransmitters transport through single live cell. Sensing techniques including electrode-based methods, optogenetics, and nanowire cell penetration systems have been developed to monitor the neuron activities. However, their biocompatibilities remain a challenge. Protein nanopores with membrane compatibility and lumen tunability provide real-time, single-molecule sensitivities for biosensing of DNA, RNA, peptides and small molecules. In this study, an engineered protein nanopore MspA (*Mycobacterium smegmatis* porin A) through site-directed mutation with histidine selectively bind with Cu^2+^ in its internal lumen. Chelation of neurotransmitters such as L-glutamate (L-Glu), dopamine (DA) and norepinephrine (NE) with the Cu^2+^ creates specific current signals, showing different transient current blockade and dwell time in single channel electrophysiological recording. Furthermore, the functionalized M2MspA-N91H nanopores have been embedded in live HEK293T cell membrane for real-time, in situ monitoring of extracellular L-glutamate translocating through the nanopore. This biomimetic neurotransmitter nanopore has provided a new platform for future development of neuron sensors, drug carrier and artificial synapse.

**Supplementary Information:**

The online version contains supplementary material available at 10.1186/s43556-021-00026-3.

## Introduction

Detecting neurotransmitter transmission between neurons is the basis for understanding the regulation of nerve signals, which is not only related to many diseases including Parkinson’s disease and depression [1, 2], but also the fundamentals for designing brain–computer interfaces. Thus, it is of great significance to develop sensors with high temporal-spatial resolution that can detect multiple neurotransmitters. Classic methods such as HPLC, MS coupled with microdialysis, are able to analyze multiple neurotransmitters with high sensitivity [3, 4]. However, sampling intervals (up to few seconds [5]) and in vitro detection are their limitations. Electrochemical techniques using nanoelectrode enable amperometric measurement inside single synapses [6], but generally only one neurotransmitter can be determined at a time [7]. Genetically encoded fluorescent sensor was used to monitor NE transmission in vivo in both physiological and pathological processes, revealing the potential of membrane protein for sensing neurotransmitters [8]. Nanopore method is proved to have the ability for the detection of multiple analytes in real-time [9]. Furthermore, new micro-nano devices and wearable/implantable diagnostic devices have been developed for the treatment of neuropathic pain, drug release and biosensing [10].

Nanopore technology has been developed for real-time, single-molecule sensing of a variety of analytes with high sensitivity and surface modification adaptability. Since α-hemolysin (α-HL) was proposed to be a nucleic acid sensor in 1996 for the first time [11], the application of biological nanopore as single molecule sensor has been greatly expanded. Concentrations of metal ions can be simultaneously quantified using an engineered α-HL [12]. For nucleic acids, techniques have been established to determine the sequence or specific structures of both DNA and RNA [13–17]. Besides, single molecule sensing of proteins is another promising application of nanopore technology [18]. Various nanopore proteins have been used for detection of peptides [19, 20], protein biomarkers [21–25], post-translational modifications [26], protein unfolding [27], and protein conformation [28]. Recently, Ouldali et al. reported the electrical recognition of the 20 proteinogenic amino acids [29], paving the way to nanopore protein sequencing. By employing site-directed mutagenesis, the detection sensitivity and specificity of biological nanopore are greatly improved. For example, cysteine replacement in α-HL lumen allowed the distinction of D-glucose and D-fructose under the mediation of boronic acids [30]; Mutation of K234 to cysteine in phi29 nanopore allowed the discrimination of ethane, thymine and benzene through disulfide linkage or transthioesterification reactions [31]; Interestingly, multiple neurotransmitters’ recognition and discrimination were achieved by chemically modifying α-HL mutant [32]. In summary, the chemical modification and introduction of adapters provides versatile strategies for small molecules detection.

Besides in vitro detection, nanopore has also been applied to cells, including drug carriers or cytotoxicity towards tumor cells [33, 34], while few researches about real-time monitoring intracellular or extracellular transport of biological molecules were reported. Carbon nanotube (CNT)-based porin was able to penetrate live cell membranes, showing a strong potential to be a biomimetic sensor for stochastic detection [35]. Comparing to synthetic channels, protein nanopores should possess better biocompatibility with cell membrane and higher possibility for molecular engineering [36]. Thus, they are preferred candidates for artificial sensors on cell membranes for monitoring intracellular or extracellular transport of biological molecules.

*Mycobacterium smegmatis* porin A (MspA), known as a homo-octameric channel for transporting hydrophilic molecules in *Mycobacterium smegmatis*, is a distinctly robust protein that retains channel-forming property at extreme pH and temperature [37]. With a short and narrow channel constriction region, MspA nanopore is a promising single-molecule analytical device. Recently, mutant MspA has been used for observing single molecule chemistry and hard-soft-acid-base interaction [38, 39]. Furthermore, MspA is utilized to assist clinical diagnosis such as detection of circulating tumor cells and rapid assay for carbapenem-resistant *Klebsiella pneumoniae* [40, 41]. Thus, MspA with site-specific mutagenesis modification holds great promise as an ideal biosensor for detecting various analytes.

In this study, we developed a neurotransmitters biosensor by site-directed mutagenesis of MspA nanopore to detect neurotransmitters real-time in single-molecule level. Furthermore, the biosensor was successfully incorporated into the membrane of single live cell for monitoring the transport of cellular neurotransmitters, which laid the foundation for future development of in situ biomimetic neurotransmitter sensor for intercellular signal transmission and neuron signaling.

## Results

### Engineering MspA nanopore protein

**Fig. 1:**
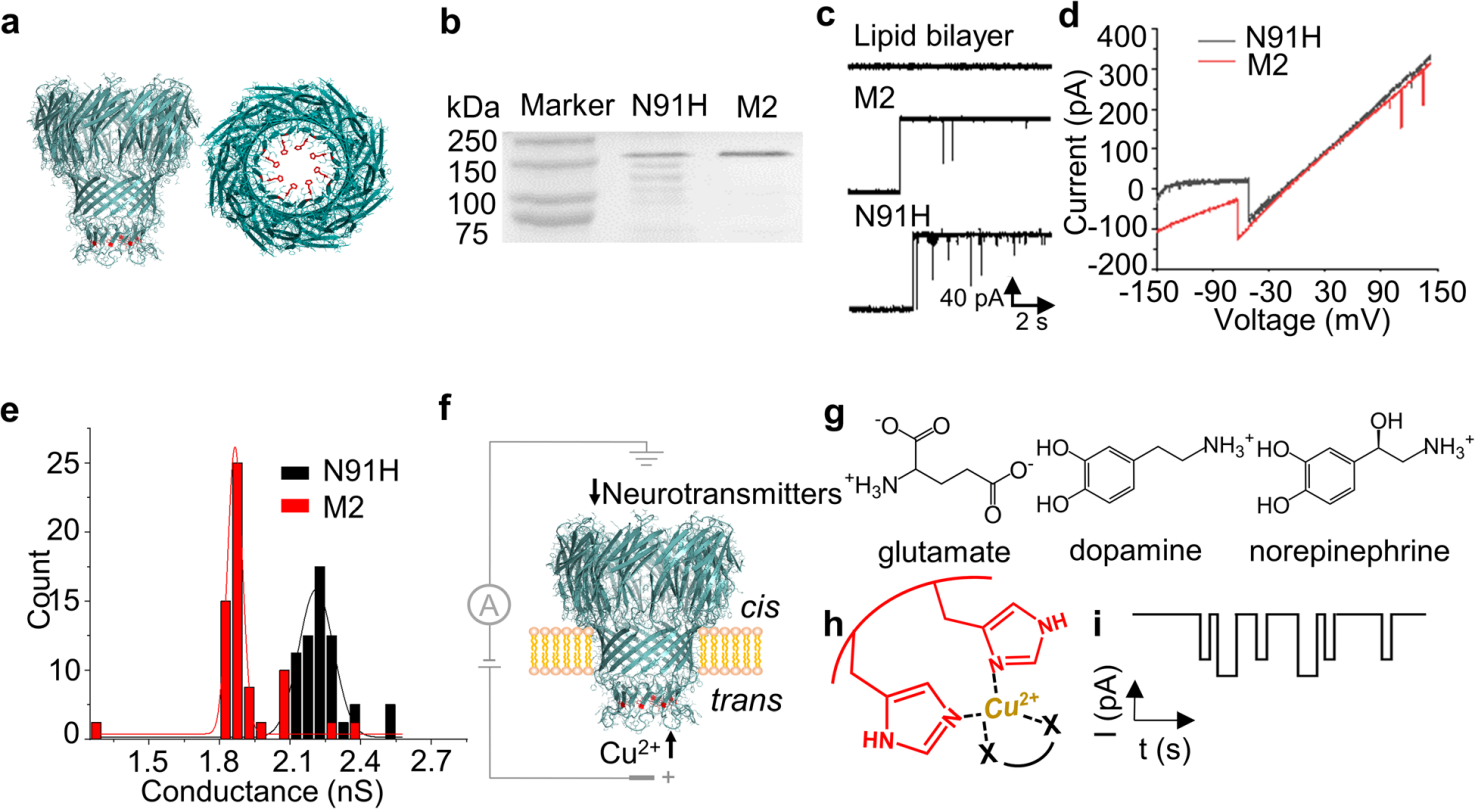
Electrophysiological properties of the engineered M2MspA-N91H and scheme of neurotransmitters detection. **a** Side-view (left) and top-view (right) of M2MspA-N91H structure modeled by SWISS-MODEL. **b** SDS-PAGE analysis of M2MspA-N91H and M2MspA. N91H represents M2MspA-N91H, and M2 represents M2MspA. **c** Typical current traces of blank, M2MspA and M2MspA-N91H nanopore inserting into lipid bilayer in 1 M KCl, pH 7.5, 50 mV. **d** I-V curves of M2MspA-N91H and M2MspA at scanning potential from − 150 to 150 mV. **e** Conductance histograms of M2MspA-N91H and M2MspA nanopore inserting into lipid bilayer in 1 M KCl, pH 7.5 (*n* = 35). **f** Experimental setup. The mutant protein M2MspA-N91H is incorporated into lipid bilayer from the *cis* (grounded) side of the setup. Cu^2+^ and neurotransmitters are added to *trans* and *cis* side, respectively, which both move towards the constriction region of M2MspA-N91H under a positive potential. **g** Ionization structures of three neurotransmitters: glutamate, dopamine and norepinephrine. **h** The principle of detection of neurotransmitters. Cu^2+^ non-covalently binds to the two nearby histidine residues at the constriction region of M2MspA-N91H firstly, then neurotransmitters which can interact with Cu^2+^ are detected. **i** Idealized current signals caused by different kinds of neurotransmitters binding reversibly to the Cu^2+^-M2MspA-N91H complex

In this study, the mutant protein M2MspA-N91H was obtained by substituting the aspartate residues at site 91 inside M2MspA lumen with histidine residues. The structure of M2MspA-N91H was predicted and modeled by SWISS-MODEL [42]. The model showed that the mutant was an octamer with eight histidine residues at the constriction region, orienting towards the central axis of M2MspA-N91H (Fig. 1a). The mutant was successfully expressed and SDS-PAGE analysis showed the purified protein had a similar molecular weight to M2MspA (Fig. 1b). Electrophysiology assay was conducted to verify the correct assembly of M2MspA-N91H. Current traces of single channel recording demonstrated that the engineered protein could be incorporated into lipid bilayer (Fig. 1c) and voltage-gating phenomenon was observed when applied trans-membrane potential was lower than − 60 mV (Fig. 1d), which was similar to the behavior of M2MspA in the same condition. The measured conductance of M2MspA and M2MspA-N91H were 1.864 ± 0.003 nS and 2.209 ± 0.006 nS in 1 M KCl, respectively (Fig. 1e).

### Oscillation of histidine residues and Cu^2+^ binding

The incorporation of single M2MspA-N91H into lipid bilayer resulted in a current increase with a fluctuation of about 30 pA around the baseline under an applied potential of 50 mV in 1 M KCl (pH 7.5) (Supplementary Fig. 1). We calculated that the change in cross-sectional area resulting in 30 pA fluctuation was about 25.3 Å^2^ (Eq. S1), which was very close to the change of cross-sectional area caused by the oscillation of eight histidine residues inside the nanopore (Supplementary Fig. 2). Combining the quite background current level of M2MspA nanopore as control, it is concluded that the fluctuations were related with the oscillation of eight histidine residues in the lumen.

**Fig. 2:**
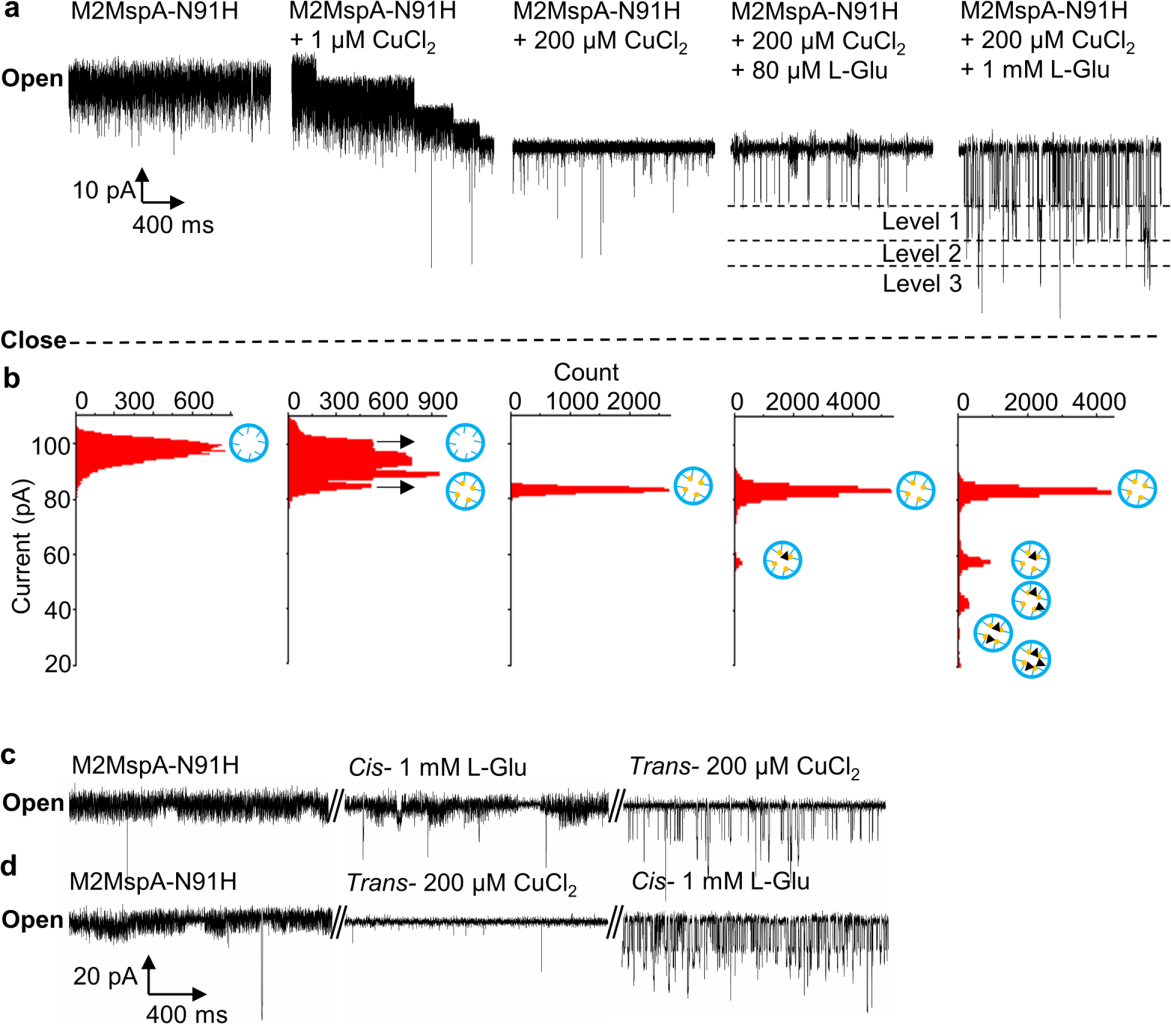
Binding of Cu^2+^ to M2MspA-N91H and L-Glu detection. **a**. Representative single-channel current traces of M2MspA-N91H open pore baseline, the addition of 1 μM CuCl_2_, 200 μM CuCl_2_ to trans chamber as well as the addition of 80 μM, 1 mM L-Glu to cis chamber. **b** All points current histogram distribution of A, the cartoon insets illustrate the possible binding numbers and positions of Cu^2+^ and L-Glu. Blue hollow circles represent M2MspA-N91H, yellow solid circles represent Cu^2+^, black solid triangles represent L-Glu. **c,d** Representative single-channel current traces of M2MspA-N91H open pore, 1 mM L-Glu and 200 μM CuCl_2_, showing different signal changes generated by adding 1 mM L-Glu and 200 μM CuCl_2_ to *trans* chamber in a different order. (1 M KCl, pH 7.5, 298 K, + 50 mV). All the experiments were repeated at least 3 times

In order to test the binding affinities of metal ions with the histidine residues inside M2MspA-N91H lumen, we added Mn^2+^, Mg^2+^, Cu^2+^, and Zn^2+^ into the *trans* chamber of the bilayer setup after a single nanopore was incorporated and observed the current traces in separate trials (Fig. 1f). Only the addition of Cu^2+^ decreased the current fluctuation significantly, while Mn^2+^, Mg^2+^, showed no binding. Zn^2+^ bound reversibly to nanopore, which was similar to the interaction between L-Glu and Cu^2+^-nanopore complex. (Supplementary Fig. 3). It was interesting to observe that the reduction of opening levels was related with the concentration of Cu^2+^ addition. One micrometre CuCl_2_ resulted in a decrease of four discrete steps of *c.a.* 7% for each step, while 200 μM CuCl_2_ induced a direct reduction of 28% of the overall opening channel, which was close to the level of four steps together at the lower CuCl_2_ concentration (Fig. 2a, b). Considering the effect of steric hindrance and eight evenly distributed histidine residues inside the lumen of M2MspA-N91H, it was proposed that one Cu^2+^ ion could bind with two adjacent histidine residues (Fig. 1h). The observed four-step reduction signal at low concentration of CuCl_2_ and stable current trace at high concentration of CuCl_2_ further indicated that the stepwise current changes were caused by Cu^2+^-histidine complex formation, and eight histidine residues in the constriction region of M2MspA-N91H could be fully coupled with four Cu^2+^ at 200 μM CuCl_2_.

### The binding of L-Glu with Cu^2+^-coupled M2MspA-N91H

Glutamate, with two carboxyl groups and one amino group, had been reported to be able to coordinate with Cu^2+^ via amine nitrogen and a carboxyl oxygen [43]. Thus, L-glutamate (L-Glu) was selected to verify the property of Cu^2+^ coupled M2MspA-N91H. In this experiment, 200 μM Cu^2+^ was chosen to guarantee the eight histidine residues inside M2MspA-N91H were fully coupled with Cu^2+^. Control experiment showed that L-Glu could not be detected without Cu^2+^ (Fig. 2c, d). With 200 μM CuCl_2_ in *trans* chamber, the addition of L-Glu to *cis* chamber generated specific binding events, demonstrating the binding of L-Glu with Cu^2+^ coupled M2MspA-N91H. Those results confirmed that both CuCl_2_ and L-Glu were dispensable for the occurrence of specific binding signals.

The blockade caused by L-Glu translocation events was about 29% (level 1 as shown in Fig. 2a) at 50 mV in 1 M KCl solution with 80 μM L-Glu, and the histogram of all points current showed a single distribution indicating only 1 L-Glu binding to the Cu^2+^ coupled M2MspA-N91H. Interestingly, four major current levels were observed at higher concentration of L-Glu (1 mM), of which the blockade was 29% (level 1), 48% (level 2), 59% (level 3) and 74% (level 4) respectively (Fig. 2a, b). These stepwise signals could be considered as sequential binding of glutamate with the Cu^2+^ coupled M2MspA-N91H. The blockade of level 3 was nearly twice of level 1, corresponding to two L-Glu binding. A blockade of 48% (level 2, 19% bigger than level 1 and 11% smaller than level 3) was also observed. Since the four-fold symmetry gave two possible relative positions for two glutamates, it could be explained that level 2 and 3 were caused by the second glutamate binding to the ortho- or para-position with respect to the first L-Glu. As Cu atom could exhibit slightly distorted square-pyramidal five-coordination with imidazole and two L-Glu, which formed a more compact structure comparing with the structure of two L-Glu binding at para-position (four-coordination). Thus, the binding of the second L-Glu in ortho-position (level 2) had a smaller blockade. Level 4 (26% bigger than level 2 and 15% bigger than level 3) showed the binding of three L-Glu. Four L-Glu binding events was not observed, which can be ascribed to the relatively low capture rate compared to the dissociation rate of the neurotransmitters and the steric hindrance. Thus, the binding dynamics between L-Glu and Cu^2+^ coupled M2MspA-N91H could be revealed and explained by the different blockades.

### Detection of L-Glu, DA and NE

**Fig. 3:**
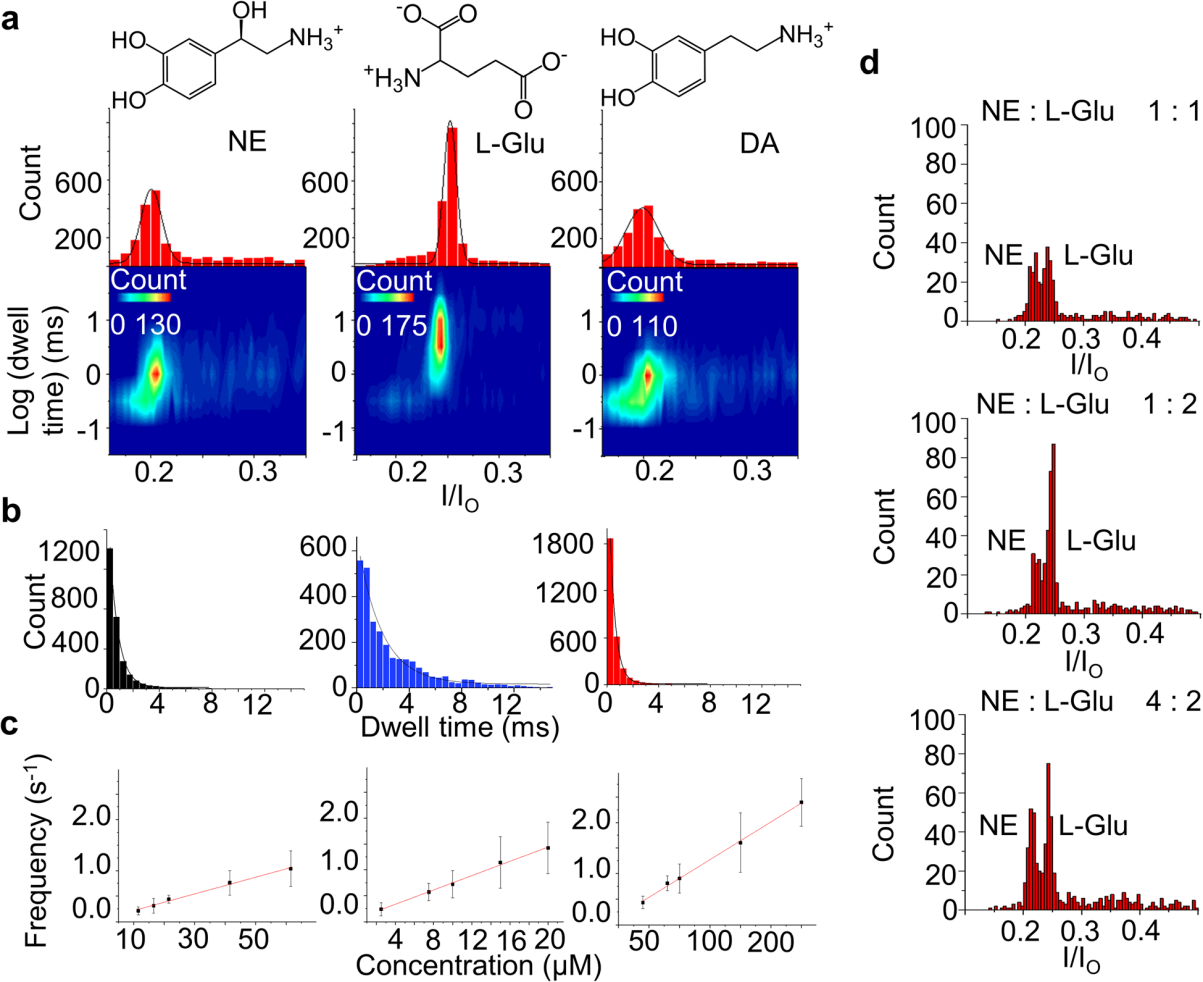
Detection of NE, L-Glu, DA by the M2MspA-N91H nanopore. **a** 2D contour plots of blockade percentage versus log (dwell time) and blockade percentage histograms of NE, L-Glu and DA from left to right, concentrations: NE, 100 μM; L-Glu, 20 μM; DA, 100 μM. **b** Event duration histograms of NE, L-Glu and DA fitted to exponential decay curves. **c** Concentration gradient experiments, showing first order dependence of event frequency on concentration of NE, L-Glu and DA (linear fit, *R*^2^ = 0.990, 0.995, 0.997). **d** Blockade percentage histograms of NE and L-Glu mix with ratio of 25 μM: 25 μM (1: 1), 25 μM: 50 μM (1: 2) and 100 μM: 50 μM (4: 2) in 50 s. (1 M KCl, pH 7.5, 298 K, + 50 mV). All the experiments were repeated at least 3 times

To verify the potential of this strategy for multiple neurotransmitters detection, three important neurotransmitters (L-glutamate (L-Glu), dopamine (DA) and norepinephrine (NE)) were tested with the engineered M2MspA-N91H nanopore (Fig. 1g, i). The analytes of interests generated specific current signals when translocating through Cu^2+^ coupled M2MspA-N91H. The scatter plots of current blockade versus log (dwell time) showed a single population of events. The current blockades caused by L-Glu, DA and NE were 25.2% ± 0.25%, 19.9% ± 0.06%, 20.1% ± 0.04% respectively, and their mean dwell time were 2.40 ± 0.22 ms, 0.45 ± 0.06 ms, 0.66 ± 0.02 ms (Fig. 3a, b, *n* = 3). The mean dwell time of L-Glu was 3–4 times longer than that of NE and DA, indicating a stronger binding ability with Cu^2+^. The translocation frequency showed a first-order dependence on the concentration of L-Glu (2 μM to 20 μM), DA (50 μM to 300 μM) and NE (10 μM to 60 μM) (Fig. 3c), which coincided with the bimolecular nature of the reaction. L-Glu showed lower limit of detection compared with NE and DA, because it was negative charged analyte while the other two were positive charged one. The sensitivity for detecting NE and DA were able to be improved by adjusting the applied potential or pH. To further assay the ability of developed sensor to distinguish different neurotransmitters in a mixture measurement, two essential neurotransmitters NE and Glu were premixed with ratio of 1:1, 1:2, and 4:2 for test. Two distributions of events in the histogram (Fig. 3d) suggested that NE and Glu could be distinguished in those conditions.

### Monitoring the transport of L-Glu through M2MspA-N91H on HEK293T cell

**Fig. 4:**
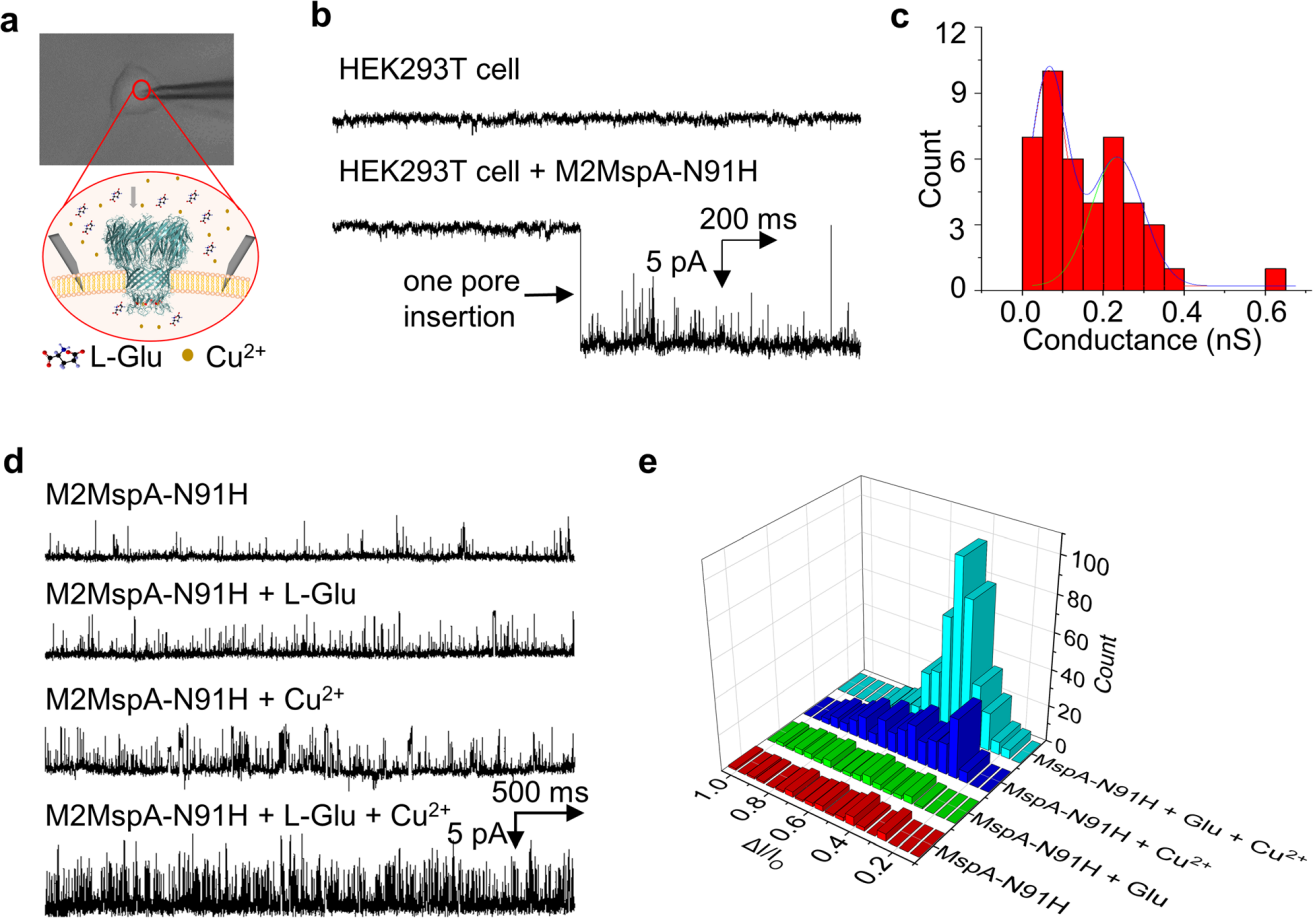
L-Glu detection by M2MspA-N91H incorporated in single live HEK293T cell membrane. **a** Glass electrode on HEK293T cell membrane (patch-mode) and schematic drawing of one MspA-91H nanopore embedded in HEK293T cell membrane. **b** Typical current traces of blank (HEK293T cell) and one M2MspA-N91H nanopore inserting into HEK293T cell membrane (HEK293T cell + M2MspA-N91H). **c** Conductance histogram of M2MspA-N91H inserting into 293 T cells (*n* = 43). **d** Typical current traces of M2MspA-N91H nanopore inserting into HEK293T cell membrane with 1 mM L-Glu and/or 200 μM Cu^2+^ at − 60 mV. **e** Current blockade 3D histogram of translocation signals from Fig. 4d. The buffer in the patch clamp pipette was 153 mM KCl, 10 mM HEPES, pH 7.3

Cell patch-clamp experiment was conducted to monitor the incorporation of M2MspA-N91H into HEK293T membrane and L-Glu transport (Fig. 4a). In the presence of M2MspA-N91H, a stepwise current signal was observed under a holding potential of − 60 mV (Fig. 4b), which was similar to the insertion signal of this protein in solution mimicking physiological condition (Supplementary Fig. 4), indicating that one M2MspA-N91H nanopore was successfully incorporated into the cell membrane. The conductance of M2MspA-N91H on HEK293T cell distributed at 0.234 ± 0.011 nS, which was calculated using the voltage of − 60 mV. In planar lipid bilayer experiments, the measured conductance was 0.36 ± 0.01 nS (150 mM KCl, *n* = 3). The inconsistency of conductance could be attributed to the effect of resting membrane potential of the cell. A small peak of 0.065 ± 0.01 nS was also observed, which could be attributed to the block by the biomolecules in the cytoplasm. Several control experiments were conducted under different conditions to verify the possibility of L-Glu detection through M2MspA-N91H on HEK293T cell. Those results showed that only in the presence of L-Glu and Cu^2+^, specific signals related with L-Glu could be observed (Fig. 4d). The peak of the L-Glu translocation blockade was around 42.20% ± 4.47% (Fig. 4e).

## Discussion

In this study, we successfully engineered a robust, functional nanopore M2MspA-N91H, which had a strong binding ability with Cu^2+^. The binding of Cu^2+^ ion decreased the current fluctuation significantly (Fig. 2a). Control experiments showed that L-glutamate could not be detected without Cu^2+^-M2MspA-N91H complex (Fig. 2c, d). This Cu^2+^ coupled M2MspA-N91H nanopore showed high sensitivity for L-glutamate, norepinephrine and dopamine translocation in BLM single channel recording. Control experiments and conformational analysis on the binding between L-glutamate and Cu^2+^ coupled M2MspA-N91H nanopore revealed details of their molecular interaction. Four major current levels were observed at a high concentration of L-Glu (1 mM), of which the blockade was 29% (level 1), 48% (level 2), 59% (level 3) and 74% (level 4) respectively (Fig. 2a, b). These stepwise signals could be considered as sequential binding of glutamate with the Cu^2+^ coupled M2MspA-N91H. Since the four-fold symmetry gave two possible relative positions for two glutamates, it could be explained that level 2 and 3 were caused by the second glutamate binding to the ortho- or para-position with respect to the first L-Glu. The different blockades revealed the number and relative position of L-Glu binding event.

Further demonstration of real time, in situ detection of the L-Glu was completed by the M2MspA-N91H nanopore incorporated in live HEK293T cell membrane. Control experiments were conducted under different conditions to verify the possibility of L-Glu detection through M2MspA-N91H on HEK293T cell. Those results showed that only in the presence of L-Glu and Cu^2+^, specific signals with blockade of 42.20% ± 4.47% could be observed (Fig. 4d), indicating the ortho-position of two L-Glu in nanopore is the most probable case in this experiment. The developed sensor shows potential in developing sensitive and portable devices for clinical diagnosis of neuron-related diseases, providing a platform for the study of neural signaling, design of artificial synapse, and development of nano-neuron interfaces.

## Material and methods

### Expression and purification of M2MspA-N91H

All mutants were obtained through site-directed mutagenesis of M2MspA [44]. A gene containing M2MspA with histidine replacement of aspartate at 91 amino acid was cloned into vector pET28b using *Nco* I and *Not* I restriction digestion sites. Then, constructed plasmid was transferred into *E. coli* BL21 (DE3) competent cell. Cells with kanamycin resistance were picked and cultured at 37 °C in lysogeny broth (LB) media containing kanamycin (50 μg/mL). One millimetre isopropyl β-D-1-thiogalactopyranoside (IPTG) was added when an optical density of cells at 600 nm reached to 0.8 to induce protein expression. Bacteria were incubated at 15 °C with 220 rpm shaking for 12 h. Cells were harvested through centrifugation at 4000 rpm, 4 °C for 15 min, then resuspended by buffer. After sonication with Ultrasonic cell disruption device, the supernatant was retained and furtherly purified by anion exchange column (Q-Sepharose) and size exclusion column (Superdex 200 16/90). 10% SDS-PAGE was used to analyze the purification result.

### Single channel recording

Nanopore experiment was carried out in a vertical setup which was separated into *cis-* and *trans-* chamber by a cup containing an aperture with a diameter of 50 μm in the middle. Ag/AgCl electrodes were inserted into each chamber. The reference electrode was connected to *trans-* chamber, and the *cis-* chamber was grounded. Both *cis-* and *trans-* sides were filled with 1 mL symmetric buffer. Lipid bilayer was formed based on the Montal-Mueller technique [45]. Briefly, the inside and outside of the aperture were pretreated with 20 mg/mL DPhPC which was dissolved in decane. After the addition of buffer, few 20 mg/mL DPhPC was applied to the above of the pretreated aperture, then the lipid bilayer was spontaneously formed by lowering the buffer below the aperture and the purified M2MspA-N91H protein was added to *cis-* side. CuCl_2_ was added to the *trans-* side when a single protein was incorporated into the lipid bilayer, and analytes of interest were added into the *cis-* chamber. Slightly pipette the solution to distribute the analytes evenly. Buffer used in planar lipid bilayer experiments was 1 M KCl (pH 7.5). All experiments were completed under an applying potential of + 50 mV. The current recording was acquired with a HEKA EPC10 patch-clamp amplifier (HEKA Elektronik, Lambrecht/Pfalz, Germany) at a sample frequency of 10 kHz. The electrophysiological experiments were carried out at 25 ± 1 °C.

### Data analysis

Data analysis was performed by using Clampfit 10.4 software filtered with a lowpass gaussian filter with a 5 kHz cutoff for data statistics. The dwell time, interval time (time between two events), blocked current (ΔI) and open pore current (I_O_) were detected through a function of single channel research. Plots including scatter diagrams, histograms and fitted curves were accomplished with Origin 9. The mean value of blocked current was obtained from the fitted Gaussian distribution of ΔI histogram. The dwell time and interval time histograms were fitted to single exponential function. All data presented were from three or more independent experiments.

### Cell culture

The human embryonic kidney 293 T cell line (HEK293T) was obtained from Chinese Academy of Sciences (Shanghai, China). The cells were cultured in DMEM supplemented with 10% FBS and incubated in a humidified atmosphere with 5% CO_2_ and 95% air at 37 °C. After reaching high density, HEK293T cells were plated in 35 mm cell dishes with an 8 mm glass coverslip.

### Cell patch-clamp experiment

A glass coverslip with HEK293T cells was placed in a cell recording chamber filled with extracellular solution containing 142 mM NaCl, 10 mM D-(+)-glucose, 8 mM KCl, 6 mM MgCl_2_·6H_2_O, 1 mM CaCl_2_, and 10 mM HEPES, pH 7.4. For cell patch-clamp experiments, each pipette was produced by a micropipette puller (P-1000, Sutter, USA) and filled with the following solution: 153 mM KCl, 10 mM HEPES, pH 7.3. Pipette tips were polished by the microforge (MF-830, Narishige, Japan) and the resistance of pipettes was in the range of 10–15 MΩ. At the beginning of each experiment, M2MspA-N91H protein, copper chloride (CuCl_2_) and glutamate solution (L-Glu) were mixed and added in pipettes according to different experimental requirements. After the formation of a giga-seal cell state recorded under − 80 mV to 20 mV holding potentials, data were obtained via Axon 700B amplifier (Molecular Devices, Axon, USA) digitized at a 50 kHz sampling rate using an AD/DA converter (Digidata 1550B, Axon, USA) with internal filter at 2 kHz.

## Supplementary Information

**Additional file 1: Supplementary Figure 1.** Current recording and current distribution of M2MspA-N91H before (a) and after (b) the addition of 200 μM Cu^2+^. (1 M KCl, pH 7.5, 298 K, +50 mV). **Supplementary Figure 2.** The model of the oscillation of histidine residues in the constriction region of M2MspA-N91H. The maximum cross-section area of the pore (left) and the minimum (right). **Supplementary Figure 3.** Binding selectivity of M2MspA-N91H to different divalent ions. (a-e) Current recording of control (a) and the addition of Mn^2+^, Mg^2+^, Zn^2+^, Cu^2+^ (b-e). The concentrations of all ions are 200 μM. (f-j) all points current histogram distribution of a-e. (1 M KCl, pH 7.5, 298 K, +50 mV). **Supplementary Figure 4.** Typical current recording traces of blank and one M2MspA-N91H nanopore inserting into lipid bilayer in 153 mM KCl, pH 7.4, mimicking physiological condition.

## Data Availability

The datasets generated during and/or analyzed during the current study are available from the corresponding author on reasonable request.

## Abbreviations

MspA: *Mycobacterium smegmatis* porin A
L-Glu: L-glutamate
NE: Norepinephrine
DA: Dopamine
HEK293T: Human embryonic kidney 293 T
HPLC: High performance liquid chromatography
MS: Mass spectrometry
α-HL: α-hemolysin
CNT: Carbon nanotube
DPhPC: Diphytanoyl phosphatidylcholine
