# Supplementary material for: Real-time sensing of neurotransmitters by functionalized nanopores embedded in a single live cell
Source: Mol Biomed. 2021 Feb 28;2:6. doi: 10.1186/s43556-021-00026-3 (PMC8607392; doi:10.1186/s43556-021-00026-3)
Supplement: Supplementary file 1 — Additional file 1: Supplementary Figure 1. Current recording and current distribution of M2MspA-N91H before (a) and after (b) the addition of 200 μM Cu2+. (1 M KCl, pH 7.5, 298 K, +50 mV). Supplementary Figure 2. The model of the oscillation of histidine residues in the constriction region of M2MspA-N91H. The maximum cross-section area of the pore (left) and the minimum (right). Supplementary Figure 3. Binding selectivity of M2MspA-N91H to different divalent ions. (a-e) Current recording of control (a) and the addition of Mn2+, Mg2+, Zn2+, Cu2+ (b-e). The concentrations of all ions are 200 μM. (f-j) all points current histogram distribution of a-e. (1 M KCl, pH 7.5, 298 K, +50 mV). Supplementary Figure 4. Typical current recording traces of blank and one M2MspA-N91H nanopore inserting into lipid bilayer in 153 mM KCl, pH 7.4, mimicking physiological condition. [file 43556_2021_26_MOESM1_ESM.docx]

**Supplemental Information**

Real-time Sensing of Neurotransmitters by Functionalized Nanopores Embedded in a Single Live Cell

**Xialin Zhang^1#^, Linqin Dou^1#^, Ming Zhang^1#^, Yu Wang^1^, Xin Jiang^1^, Xinqiong Li^1^, Long Wei^1^, Yuejia Chen^1^, Cuisong Zhou^2^, and Jia Geng^1*^**

^1^ Department of Laboratory Medicine, State Key Laboratory of Biotherapy and Cancer Center, West China Hospital, Sichuan University and Collaborative Innovation Center, Chengdu, 610041, China

^2^ College of Chemistry, Sichuan University. Chengdu, 610041, China

**Corresponding Author:** geng.jia@scu.edu.cn **(**J.G)

#These authors contributed equally to this work


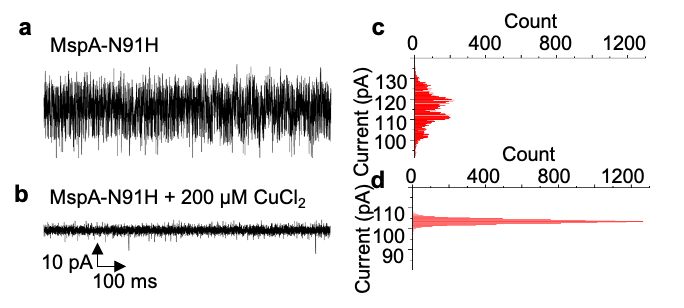


**Supplementary Fig.1** Current recording and current distribution of M2MspA-N91H before (a) and after (b) the addition of 200 μM Cu^2+^. (1 M KCl, pH 7.5, 298 K, +50 mV)


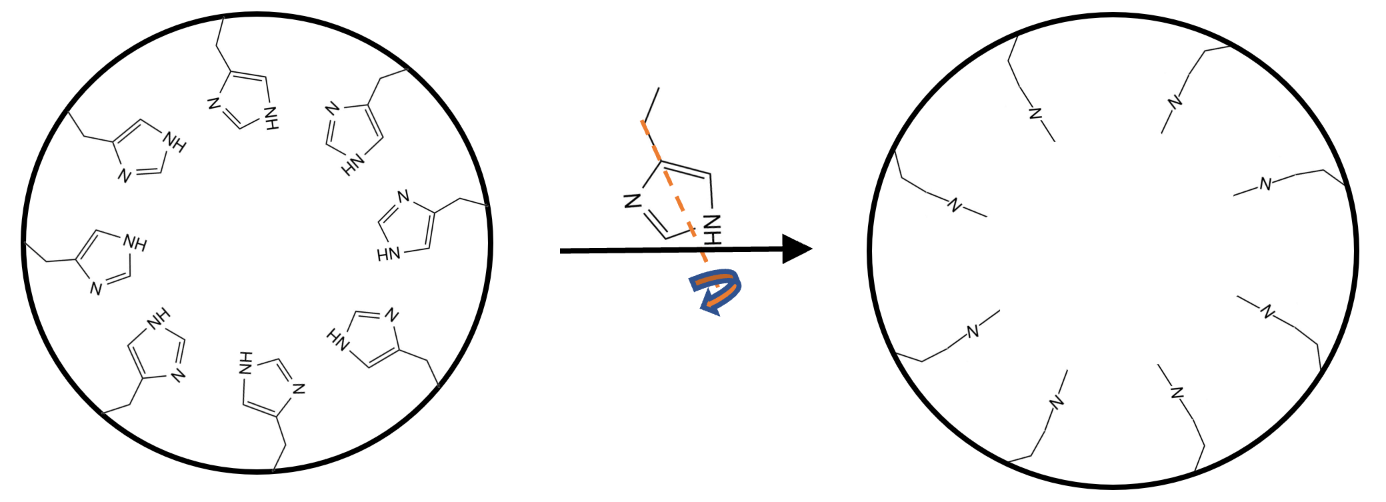


**Supplementary Fig.2** The model of the oscillation of histidine residues in the constriction region of M2MspA-N91H. The maximum cross-section area of the pore (left) and the minimum (right).


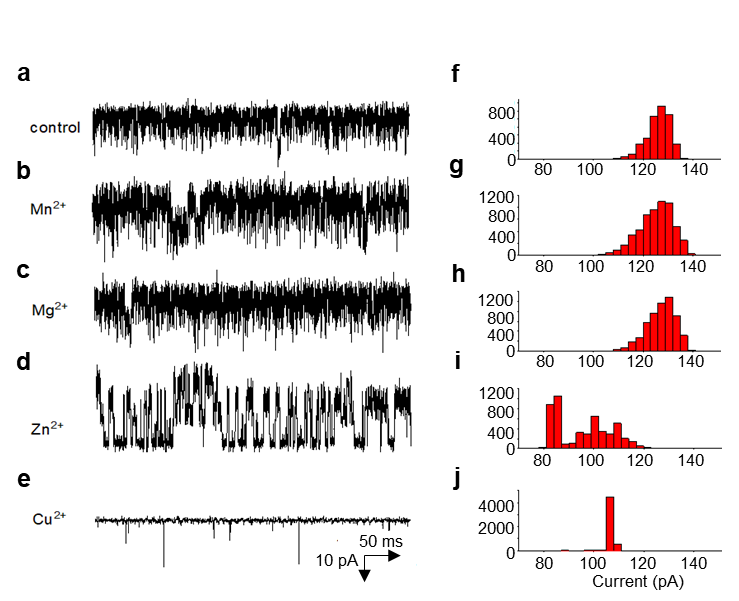


**Supplementary Fig.3** Binding selectivity of M2MspA-N91H to different divalent ions. (a-e) Current recording of control (a) and the addition of Mn^2+^, Mg^2+^, Zn^2+^, Cu^2+^ (b-e). The concentrations of all ions are 200 μM. (f-j) all points current histogram distribution of a-e. (1 M KCl, pH 7.5, 298 K, +50 mV)





**Supplementary Fig.4** Typical current recording traces of blank and one M2MspA-N91H nanopore inserting into lipid bilayer in 153 mM KCl, pH 7.4, mimicking physiological condition.

Equation △Conductance/Conductance of M2MspA = △S/πR^2^ = 0.6 nS/1.86 nS (S1)

△Conductance represents the conductance change caused by the 30 pA fluctuation under a voltage of 50 mV in 1M KCl (pH 7.5). The diameter of constriction region M2MspA was reported as 10 Å (Butler et al., 2008). (R = 5 Å)

The calculated △S is 25.3 Å^2^, which means a 25.3 Å^2^ cross-section area change result in the 30 pA fluctuation.

Calculated area of one imidazole using AutoCAD is 3.4002 Å^2^. Area of eight imidazole groups is: 8×*S*_imidazole_ = 8×3.4002 Å^2^ = 27.2 Å^2^

The change of cross-section area caused by the oscillation of eight histidine residues is approximately 27.2 Å^2^, which is quite close to the △S in equation S1, indicating that the current fluctuation results from the oscillation of eight histidine residues.

References

Butler, T.Z., Pavlenok, M., Derrington, I.M., Niederweis, M., Gundlach, J.H., 2008. Single-molecule DNA detection with an engineered MspA protein nanopore. Proc. Natl. Acad. Sci. U. S. A. 105, 20647–20652. https://doi.org/10.1073/pnas.0807514106
